# A novel study to calculate immune-aging from peripheral blood T lymphocyte subsets and their mitochondrial parameters in healthy Chinese subjects

**DOI:** 10.3389/fimmu.2026.1857636

**Published:** 2026-07-07

**Authors:** Guofang Gan, Peng Guo, Xufan Li, Sihan Zhang, Wei Zhao, Yu Gao, Zhenchao Zhuang

**Affiliations:** 1 Clinical Laboratory, Huzhou Maternity & Child Health Care Hospital, Huzhou, China; 2 UBBio Technology (Zhejiang) Co., LTD, Hangzhou, China; 3 Key Laboratory of Biomarkers and In Vitro Diagnosis Translation of Zhejiang Province, Zhejiang, Hangzhou, China; 4 School of Computer Science, Wuhan University, Wuhan, China; 5 Su Zhou AI-For-Cure Company, Suzhou, China; 6 Zhejiang Hospital, Department of Hematology, Hangzhou, China; 7 Adicon Clinical Laboratories, Hangzhou, Zhejiang, China; 8 Department of Laboratory Medicine, The First Affiliated Hospital of Zhejiang Chinese Medical University (Zhejiang Provincial Hospital of Chinese Medicine), Hangzhou, China

**Keywords:** machine learning, mitochondrial mass, mitochondrial membrane potential, potential markers of immunosenescence (PMIS), T lymphocyte subsets

## Abstract

**Background:**

Immunosenescence is a process in which the body’s immune function declines with age, which is associated with the increased risk of infection, tumors, and other diseases. Traditional biological age assessment is difficult to fully reflect the changes in immune function. Flow cytometry can obtain multi-dimensional data such as immune cell subsets and mitochondrial function, and combined with machine learning, it is possible to quantify immune-aging. This study aimed to establish an accurate immune-aging prediction model based on peripheral blood indicators of healthy people in China.

**Methods:**

Peripheral blood samples were collected from healthy people aged 0.5 to 89 years old from September 2023 to December 2023, and 72 indicators (including blood routine, biochemistry, T/B/NK cell subsets, mitochondrial mass, and percentage of low membrane potential, etc.) were detected. A total of 11 core features were obtained by multi-stage screening of automatic and manual feature construction and mutual information-Boruta-forward selection. Random forest, LightGBM, and other algorithms were used, combined with the Bagging fusion strategy for modeling, and cancer patients were used as the external validation set.

**Results:**

The LightGBM model had the best performance, with an R² of 0.809 and an MAE of 5.687 in the test set of the healthy population. The immune-aging of cancer patients was significantly higher than the actual age. The model had a strong discrimination (AUC>0.900) in the adolescent group (0–19 years old) and the elderly group (≥60 years old), and a slightly weaker discrimination (AUC>0.780) in the middle-aged group.

**Conclusion:**

This model can accurately quantify the degree of immune senescence, provide reference for health management, vaccination, and anti-aging intervention, and also provide new ideas for the judgment of aging-related diseases.

## 1 Introduction

The immune system is an important defense line to resist pathogen invasion, remove abnormal cells, and maintain homeostasis. With aging, the function of the immune system gradually declines, and this process is known as immunosenescence. Immunosenescence is characterized by changes in the composition and function of immune cells and increased levels of chronic inflammation. It not only increases the risk of infection, malignancies and autoimmune diseases, but also is closely related to weakened response to vaccination and reduced recovery ability (1–3).

Traditional biological age assessment mostly relies on clinical chemical indicators, telomere length, or epigenetic clock, which can reflect the aging state of the body to a certain extent, but often cannot fully reveal the functional changes of the immune system (4–6). Furthermore, the phenotypic characteristics of immune system aging often show high-dimensional, multivariate, and nonlinear relationships, and traditional single-indicator or linear models are difficult to accurately capture these complex patterns. And the immune system may be damaged to varying degrees due to factors such as lifestyle, environment, and genetics, resulting in differences in the degree of immune decline among individuals of the same age group (7, 8). Therefore, precise monitoring is necessary to study the progressive changes that occur in the immune system during the aging process.

Potential markers of immunosenescence (PMIS) is used to quantitatively characterize the degree of aging of an individual’s immune system (9). Given that chronological age is the most stable and significant driver of immunosenescence, it can be used as the target variable of supervised learning to guide the model to learn the law of immune system changes with age from immunological characteristics, and the predictive value of the model can be used as the PMIS of individuals.

In recent years, the development of flow cytometry allows researchers to simultaneously obtain multi-dimensional data, such as the proportion of immune cell subsets, the functional status of mitochondria (such as mitochondrial membrane potential, mitochondrial mass), as well as the expression of the PD-1 immunosuppressive molecules (10, 11). This makes it possible to construct a more accurate quantitative model of immunosenescence. Meanwhile, machine learning technology (ML) has the advantages of automatic feature selection and nonlinear modeling, which can dig out the core combination of immunosenescence from high-dimensional immunological data, and provide a new solution for predicting the “immune age” of individuals (12).

Quantification of PMIS can be used to assess individual immune function and guide vaccination strategies, immune rehabilitation programs, and anti-aging interventions. In this study, we developed a novel model based on peripheral blood indicators to predict the PMIS of the healthy Chinese population. This model can track the changes of PMIS, dynamically evaluate the effectiveness of health interventions, and achieve precise health management.

## 2 Materials and methods

### 2.1 Study population

During September 2023 to December 2023, we obtained the valid immune cell data of 741 samples of healthy Chinese people aged 0.5 to 89 years old (including monocytes, lymphocytes and their subsets of T cells, B cells, NK cells), and the different developmental stages of Th cells and Tc cells (including Tn cells, Tcm cells, Tef cells, Tem cells) and their corresponding mitochondrial parameters (including mitochondrial mass and percentage of low mitochondrial membrane potential) by flow cytometry. The detection antibody was a T cell differentiation subgroup detection kit developed and produced by UB Biotechnology (Zhejiang) Co., LTD. (Chinese Registration number: Xiang Ji Zhuzhun 20222401162, by National Medical Products Administration). NovoCyte 2060R produced by Agilent Bio (USA) was used.

### 2.2 Flow cytometry analysis and mitochondrial parameter determination

The mitochondrial parameter comprised MM and MMP^low^ (%), which were acquired with mitochondrial stain MitoDye-APC (C_34_H_36_Cl_2_N_2_, CN202110570964) by flow cytometer (DxFLEX, Beckman Coulter, USA). It had two panel flow cytometry analyses to be used (UBBio technology, Hangzhou, China): a) Lymphocyte subset mitochondrial parameter (TBNK-mito panel, short mark “T” in the parameter), including CD3/PE, CD4/PE-Cy7, CD8/FITC, CD19/FITC, CD56/PE, CD45/PerCP-Cy5.5. b) The differentiations of T cell subsets mitochondrial parameter (TFUN-mito panel, short mark “(F)” in the parameter), including CD4/FITC, CD45RA/PerCP-Cy5.5, CD62L/PE-Cy7, MitoDye/APC, CD8/APC-Cy7.

#### 2.2.1 Sample acquisition

2–5 mL of venous blood was collected in an EDTA anticoagulant tube. The blood samples were mixed gently and immediately after collection to prevent clotting and avoid violent mixing. Verify the samples were free of clots and hemolysis, and the barcodes and basic information were accurate. Samples were tested as quickly as possible and were stored at room temperature for no more than 48 h.

#### 2.2.2 Sample preparation protocol

a) 100 μL peripheral blood samples with EDTA anticoagulants were incubated with the mixed antibodies at room temperature in the dark for 15 min, and 2 mL of hemolysin was added to destroy the erythrocytes. b) The samples were centrifuged at 300× g for 5 min. c) The supernatant was discarded, and the precipitate was resuspended in 200 μL PBS, transferred to an account containing MitoDye, and incubated at 37 °C in the dark for 30 min. d) It was eventually transferred to a flow tube, where the tagged immune cells were then counted by flow cytometry. Besides, the final analysis and graphical output were performed using NovoExpress software (Agilent Technology, USA). The gating strategy was agreed upon before data analysis. Side scatter area (SSC-H) versus cell viability marker (NIR Flour/APC-Cy7) plots were used to exclude dead cells. The percentages of each cell subpopulation relative to lymphocytes, and the MM parameters were measured and calibrated to export by the human lymphocyte mitochondrial function analysis system (UBBio technology, Hangzhou, China).

### 2.3 Statistical analysis

In order to explore the linear association between the independent variable (immune index) and the dependent variable (age), Pearson’s correlation coefficient (PCC) was used for statistical analysis. This analysis was used to evaluate the correlation strength of each immunological feature with age and provide a reference for subsequent feature screening and model construction. In addition, we further calculated the PCC in different age strata to explore the variation pattern of immune indicators within specific age groups, so as to assist in explaining the performance differences of the model among different populations. The paired Wilcoxon signed-rank test was used to compare the MAE differences in 10-fold cross-validation between LightGBM and linear regression, LASSO, and decision trees. And a *p* < 0.05 was determined as having a significant statistical difference.

### 2.4 Elementary analysis

The Interquartile Range (IQR) method was used to detect outliers of immunological indicators (outliers defined as samples outside [Q_1_-1.5×IQR, Q_3_+1.5×IQR]). Considering biological heterogeneity, individual differences of immune indicators, and tree models’ insensitivity to outliers, outliers were retained to avoid losing biologically significant signals. Only non-numeric (Nan), infinite (Inf), zero, and unreasonable negative values were screened: no Nan, Inf or negative values were found, but zero values existed in 9 indicators (“T4Tef MMP%” (8 samples), “T8Tcm.PD-1^+^%” (2 samples), and “T4Tem.PD-1^+^” % “(2 samples),” T8Tef% “(2 samples),” T8Tef.MM (F) “(1 sample),” T8Tef.PD-1^+^% “(1 sample),” T8Tef MMP% “(1 sample) and” T8Tcm.MMP% “(1 sample)), which may reflect individual immune status differences and were reasonably processed in subsequent steps to ensure data integrity. Note that a positive minimal constant of 1×10–^8^ was uniformly added to all variables before log transformation (ln) to prevent undefined mathematical errors.

### 2.5 Feature construction

During the feature engineering phase, an integrated strategy of automated and manual feature construction was employed to fully exploit the potential information embedded in immunological features. Initially, Feature-Tools, which is built on the Deep Feature Synthesis (DFS) algorithm (13), was utilized for automated feature generation. The core mechanism of DFS involves recursively combining primary features to capture nonlinear relationships and interactions among variables, thereby generating higher-order features through arithmetic operations (addition, subtraction, multiplication, and division) performed on the original dataset. For the 72 original immunological features, single-layer deep feature synthesis is completed based on four basic arithmetic operations. The features were first grouped into four categories (blood routine indicators, cell mitochondrial mass, cell proportion, and cell mitochondrial low membrane potential proportion), resulting in the generation of 4458 candidate features. Notably, to mitigate the impact of extreme values on feature distribution and reduce the instability caused by numerical scale differences, logarithmic transformation was applied prior to multiplication and division operations, which were then converted into additive and subtractive forms.

On the basis of automated feature generation, manual feature construction was further carried out by incorporating prior knowledge in the field of immunometabolism. Relevant studies have demonstrated that mitochondrial membrane potential (MMP) is an indicator of cellular metabolic activity and stemness (14), while Programmed death-1 (PD-1) signaling plays a crucial regulatory role in T cell metabolic reprogramming, influencing key metabolic processes including glycolysis, lipolysis, and fatty acid oxidation (15–17). Guided by these biological mechanisms, we artificially designed multi-indicator interaction features—such as combinations of cell subset proportion, mitochondrial function indices, and PD-1 expression levels—to explore their combined effects on age-associated changes in immune function. Nevertheless, model evaluation results indicated that these domain knowledge-based higher-order manual features did not exert a significant improvement on the overall prediction performance of the model.

### 2.6 Feature selection

To improve the model’s generalization ability and reduce redundant feature interference, this study adopted a multi-stage feature screening strategy integrating the Mutual Information (MI) algorithm, Boruta algorithm, and Forward Selection method to progressively screen key features that significantly contribute to the target variables. Based on mutual information theory (18), we calculated the mutual information between each feature and the target variable to measure their statistical dependence—MI can capture both linear and nonlinear relationships, allowing us to preliminarily retain high-information variables by selecting features with high mutual information values. Subsequently, the Boruta algorithm (19) was used for further screening: taking random forest as the core model, it constructs “Shadow Features” (control variables generated by random permutation) for each feature. Through multiple rounds of iteration, the algorithm compares the importance distribution of original features and their shadow features, retaining variables that are statistically significantly better than shadow features to ensure the selected features have a stable and significant impact on the target variables. Finally, 11 optimal features co-screened by Boruta and Forward Selection were identified for subsequent modeling.

### 2.7 Machine learning models and applications

**Figure 1:**
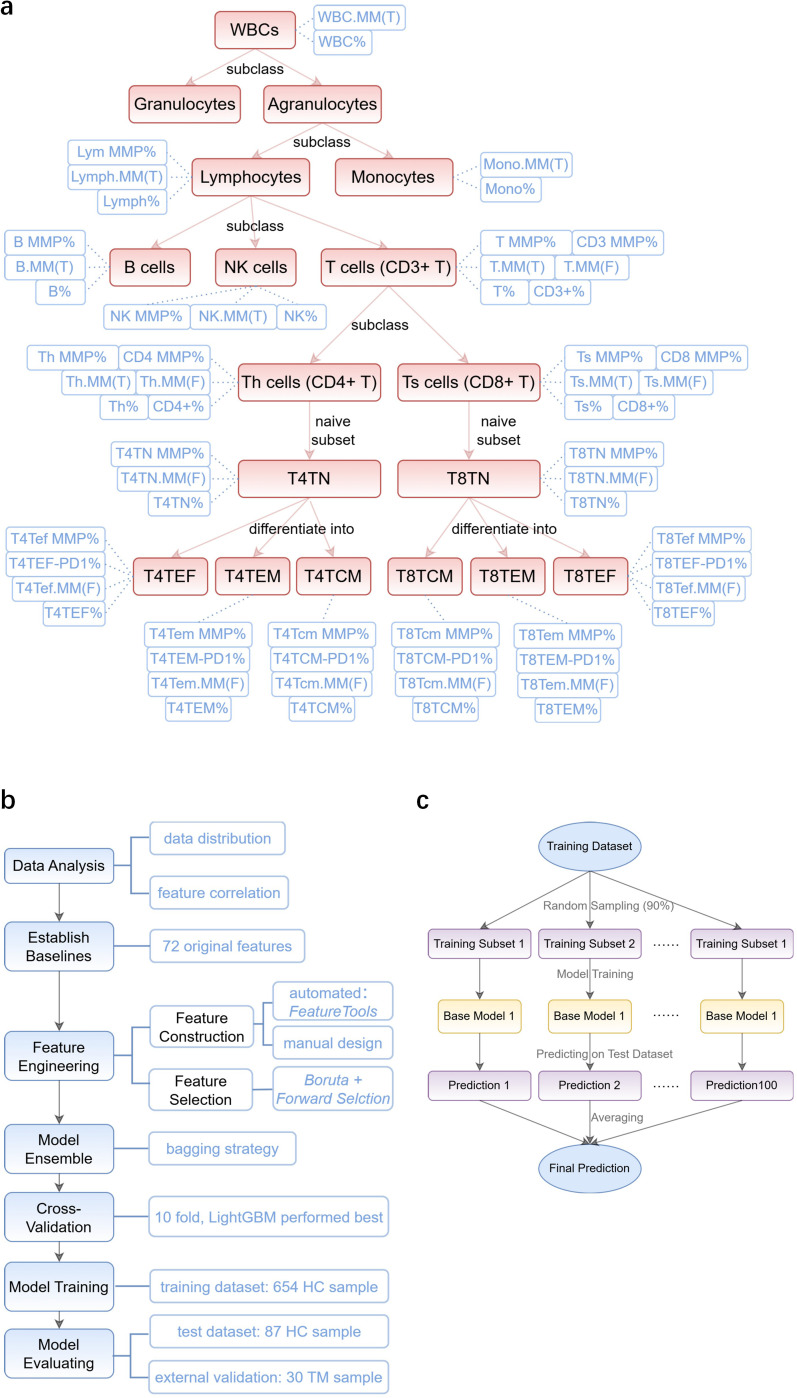
Data structure and machine learning modeling framework for PMIS prediction research. **(a)** Hierarchical schematic of immune cell populations and corresponding immunological indicators. Each cell subset is linked to quantitative metrics including subset proportion, mitochondrial mass (MM), and MMP%, (also MMP-^Low^% or MMP(^low^)%) low-mitochondrial membrane potential percentage. **(b)** Overall workflow for PMIS modeling, covering data preprocessing, feature engineering, feature selection, model training, cross-validation, ensemble optimization, and external validation. **(c)** Schematic of the Bagging ensemble strategy: 100 bootstrap samples (90% of training data) are used to train base learners, and final predictions are averaged to improve stability and generalization. PMIS, potential markers of immunosenescence; CV, cross-validation; HC, healthy control; TM, tumor patient.

In this study, machine learning algorithms were integrated to construct PMIS prediction models, with systematic evaluation of their performance and interpretability. The models included Random Forest (RF), LightGBM, XGBoost, KNN, and SVM (20–23); the full modeling and optimization workflow is illustrated in **Figure 1**. A total of 771 samples were analyzed, each comprising age, 72 immune indicators, and basic information. The dataset was split into a normal population (741 cases) and a tumor patient population (30 cases), with tumor samples serving as the external validation set. Data partitioning was stratified by four age groups (juvenile: <20 years, young adult: 20–39 years, middle-aged: 40–59 years, elderly: ≥60 years) before random selection of test data. For normal population data, 654 samples formed the training set and 87 (12%) the test set.

Baseline evaluation of the five models used raw data via cross-validation, fitting, and testing to establish benchmarks for subsequent optimization. Feature engineering combined automated and biologically informed manual construction to generate an extended feature set. Preliminary screening integrated the Boruta algorithm and Mutual Information, followed by Forward Selection to refine the optimal subset while maintaining or improving model R². Selected features were used to retrain and evaluate the five models. The SHAP method quantified each feature’s marginal contribution to model outputs (24). Global and local SHAP analyses identified key predictors of PMIS and validated their biological plausibility. Model optimization employed Bagging for ensemble learning, enhancing generalization and stability (25). The training set was resampled 100 times (90% samples per subset) to train 100 base models; final predictions were averaged. To assess performance across populations, the PMIS regression task was converted to multi-classification by stratifying samples into four age groups. Model discriminative power was evaluated via age-group-specific AUC and ROC curves. For low-AUC groups, underlying biological and distributional causes were explored using feature-age Pearson correlations and age-dependent SHAP patterns.

## 3 Results

### 3.1 Population cohort

**Figure 2:**
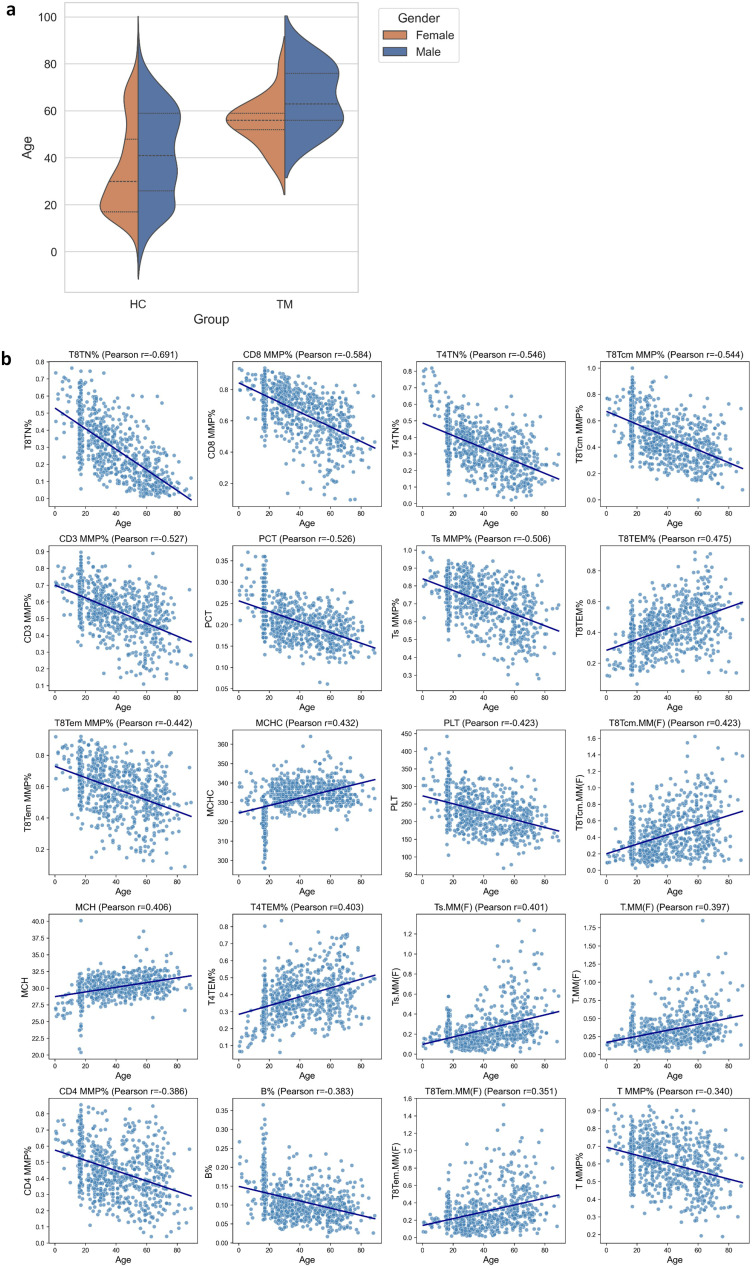
Age distribution of the study population and correlation analysis between immune indicators and age. **(a)** Violin plot of sample age distribution in the HC group versus the TM group. **(b)** The figure showed the scatter plots of the top 20 indicators with the most significant correlation with age, respectively, and the overall trend of the trend line changing with age is fitted, and the *r* value is the correlation coefficient. HC, healthy control; TM, tumor patient; T8T_N_, CD8^+^ naive T cells; T4T_N_, CD4^+^ naive T cells; T8Tcm, CD8^+^ central memory T cells; T8Tem, CD8^+^ effector memory T cells; MMP%, (also MMP-^Low^% or MMP(^low^)%) low-mitochondrial membrane potential percentage; MM, mitochondrial mass; PLT, platelet count; MCH, mean corpuscular hemoglobin; B%, B cell proportion.

The study enrolled a healthy physical examination cohort aged 0.5–90 years (both sexes) with no diagnosed diseases, totaling 741 healthy controls (HC). An independent set of 30 cancer patients (TM) served as the validation cohort. HC samples were predominantly from adolescents around 20 years old, with very low representation among individuals over 80 years. Conversely, the 30 TM samples were mainly from middle-aged and elderly individuals around 60 years old, with minimal samples under 40 years. Significant age-structural disparities existed between the two groups (**Figure 2a**).

### 3.2 Multiple indicators were strongly correlated with age

Pearson’s correlation analysis was conducted to assess associations between immune parameters and age, with a correlation coefficient threshold of 0.3 defined as significant.

Proportions of T4Tem and T8Tem cells exhibited strong positive correlations with age (r=0.403 and r=0.475, respectively). In contrast, proportions of T4Tn, T8Tn, and B cells were significantly negatively correlated with age (r=-0.546, r=-0.691, and r=-0.383, respectively). Mitochondrial mass of most immune cell types correlated positively with age, with coefficients of 0.397 (T cells), 0.423 (T8Tcm cells), and 0.401 (Ts cells). The proportion of cells with low mitochondrial membrane potential showed negative age correlations, particularly in CD3, CD8, T8Tcm, and T8Tem cells (r=-0.527, r=-0.584, r=-0.544, and r=-0.442, respectively). Among routine blood parameters, mean corpuscular hemoglobin (MCH, r=0.406), mean corpuscular hemoglobin concentration (MCHC, r=0.432), platelet hematocrit (PCT, r=-0.526), and platelet count (PLT, r=-0.423) correlated significantly with age (**Figure 2b**).

Remaining parameters, including B cell low mitochondrial membrane potential proportion, hematocrit (HCT), lymphocyte/monocyte proportions, NK cell mitochondrial mass/low membrane potential, red blood cell count (RBC), T4Tef cell proportion, and white blood cell count (WBC), showed negligible age correlations (r≈0). These findings implied that immune traits collectively reflect age-related changes via nonlinear or interactive patterns. Thus, feature combination and interaction construction were applied to explore complex immune relationships and enhance the model’s performance in predicting the PMIS index.

### 3.3 Analysis of feature screening results

**Figure 3:**
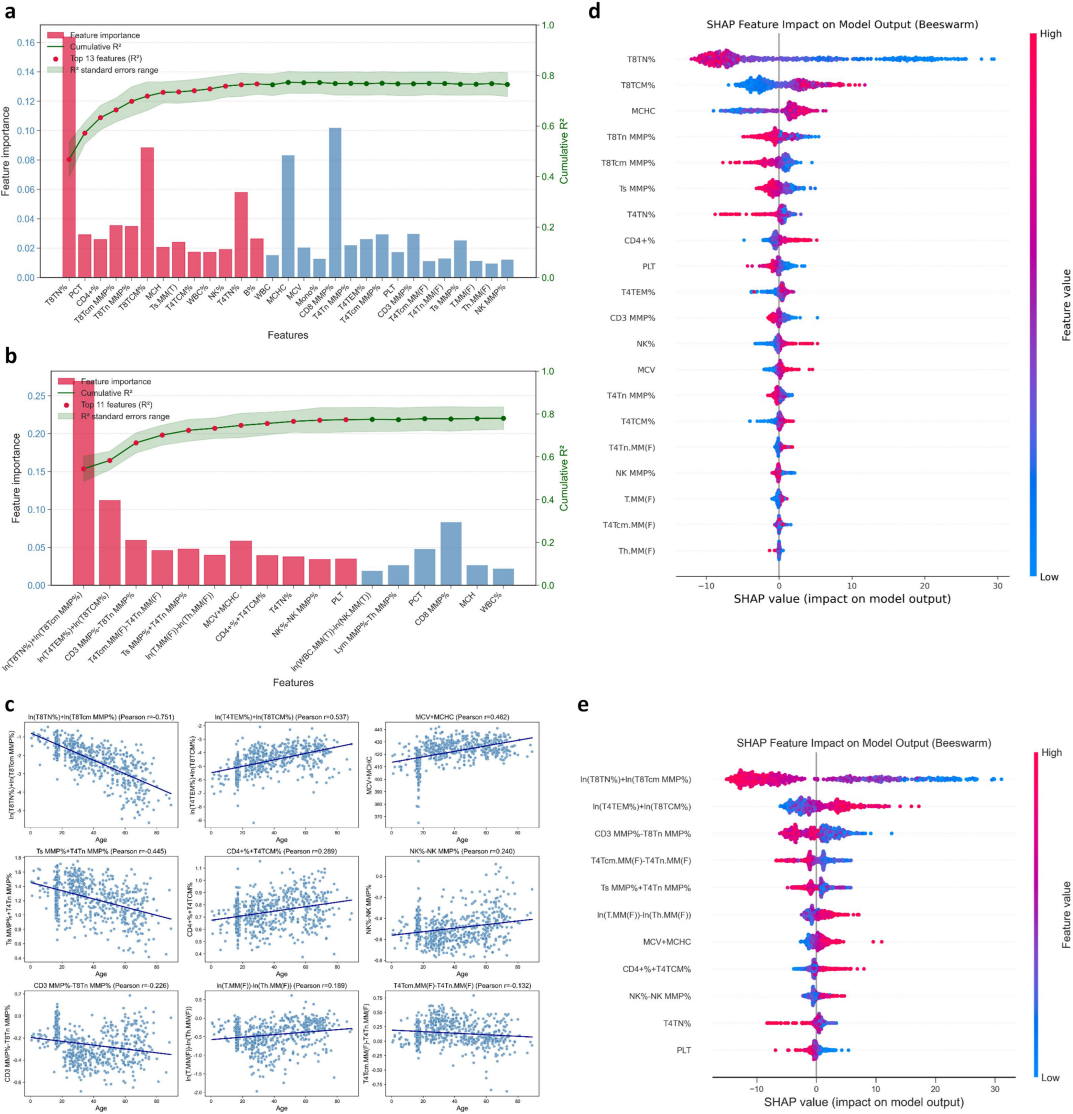
Feature screening, model performance, and SHAP-based interpretability. **(a)** Relative importance and cumulative R² of the 13 optimal raw features selected by Boruta and forward selection. **(b)** Relative importance and cumulative R² of the 11 final core combined features. **(c)** Correlation between 9 key combined features and age, with Pearson correlation coefficients. **(d)** SHAP beeswarm plot for the 20 original features underlying the 11 core features. **(e)** SHAP beeswarm plot for the 11 core features, showing direction and magnitude of feature impact on PMIS prediction (red=high feature value, blue=low feature value). SHAP, SHapley Additive exPlanations; R², coefficient of determination; PMIS, potential markers of immunosenescence.

Feature screening revealed that model R² increased with the number of input features (**Figure 3a**). The determination coefficient stabilized at approximately 0.766 after incorporating 13 original features: T8Tn%, PCT, CD4^+^%, T8Tcm.MMP%, T8Tn.MMP%, T8Tcm%, MCH, Tc.MM (T), T4Tcm%, WBC%, NK%, T4Tn%, and B%. Additional features yielded minimal performance gains, indicating these 13 features captured the core information for PMIS prediction, while subsequent features introduced redundancy or noise. Balancing model parsimony and predictive performance, these 13 features were identified as key predictors.

Screening of original features and their deduplicated combined derivatives showed that model R² stabilized at ~0.773 with 11 core features (**Figure 3b**), including: “ln(T8Tn%)+ln(T8Tcm.MMP%)”, “ln(T4Tem%)+ln(T8Tcm%)”, “CD3.MMP%-T8Tn.MMP%”, “T4Tcm.MM(F)-T4Tn.MM(F)”, “Tc.MMP%+T4Tn.MMP%”, “ln(T.MM(F))-ln(Th.MM(F))”, “MCV+MCHC”, “CD4^+^%+T4Tcm%”, “T4Tn%”, “NK%-NK.MMP%”, and “PLT”. Further feature addition did not improve R², confirming these 11 features as the model’s core set.

It is important to distinguish three feature sets used throughout this study. (i) The 13 features above are the optimal subset obtained when Boruta and forward selection are applied directly to the 72 raw features; they define the performance ceiling achievable without feature engineering (**Figure 3a**) and identify the core biological signal. (ii) The 11 core features are the final modeling set, in which biologically grouped raw variables are combined into higher-order interaction terms. (iii) The 20 features evaluated in **Table 1** are the raw constituents that underlie these 11 engineered terms (e.g. “MCV+MCHC” decomposes into MCV and MCHC), and are included as a like-for-like control that isolates the added value of the engineered interactions rather than of the underlying variables themselves. The 13-feature raw baseline and the 20-feature decomposition therefore answer different questions and are not competing descriptions of the same set.

**Table 1:** Performance of five machine learning models with three feature sets.

ML model	Features	10-fold cross-validation (train dataset, *n* = 654)				HC test dataset (*n* = 87)					TM test dataset (*n* = 30)			
		MAE	MSE	RMSE	R^2^	MAE	MSE	RMSE	R^2^	MAE		MSE	RMSE	R^2^
Rand-om Forest	Original	7.488	101.702	10.000	0.731	6.916	74.534	8.633	0.758	12.652		225.012	15.000	-0.502
	20 Features	7.302	97.840	9.828	0.740	6.876	75.597	8.695	0.754	11.326		189.711	13.774	-0.266
	11 Features	**6.674**	**87.381**	**9.264**	**0.768**	5.850	64.916	8.057	0.789	12.396		243.396	15.601	-0.625
Light-GBM	Original	7.143	92.608	9.565	0.754	6.983	79.157	8.897	0.743	12.736		217.994	14.765	-0.455
	20 Features	7.296	94.506	9.661	0.750	6.985	73.665	8.583	0.761	13.240		237.911	15.424	-0.588
	11 Features	7.097	96.937	9.774	0.742	**5.687**	**60.387**	**7.771**	**0.804**	13.795		258.818	16.088	-0.727
XGB-oost	Original	7.480	107.620	10.306	0.715	7.268	86.447	9.298	0.719	12.413		209.944	14.489	-0.401
	20 Features	7.887	111.824	10.545	0.703	6.775	79.961	8.942	0.740	11.299		178.499	13.360	-0.191
	11 Features	7.220	104.101	10.107	0.723	6.016	67.467	8.214	0.781	11.768		219.653	14.821	-0.466
KNN	Original	13.535	298.094	17.218	0.205	11.325	212.351	14.572	0.310	17.776		532.549	23.077	-2.555
	20 Features	13.219	285.560	16.878	0.238	11.187	219.090	14.802	0.288	15.387		390.048	19.750	-1.603
	11 Features	12.817	268.417	16.343	0.285	11.192	222.119	14.904	0.278	12.487		275.236	16.590	-0.837
SVM	Original	15.336	344.549	18.520	0.090	13.501	281.897	16.790	0.084	23.429		718.735	26.809	-3.797
	20 Features	15.278	341.967	18.450	0.097	13.516	281.935	16.791	0.084	22.583		679.286	26.063	-3.534
	11 Features	15.408	347.857	18.609	0.081	13.619	286.416	16.924	0.070	23.171		700.990	26.476	-3.679

The table shows the different numbers of features in the three kinds of data. Original represents the original 72 features, 11 features are the combination of original features and combined features obtained by feature engineering screening, and 20 features are all the corresponding original features involved in 11 features.

For the 11 core features, logarithmic combination of T8Tn% (r=-0.691) and T8Tcm.MMP% (r=-0.544) into “ln(T8Tn%)+ln(T8Tcm.MMP%)” strengthened the age correlation to r=-0.751 (**Figure 3c**). Combining T4Tem% (r=0.403) and T8Tcm% (r=0.286) into “ln(T4Tem%)+ln(T8Tcm%)” increased the correlation to r=0.537. This multiplicative interaction enhanced linear relationship representation, aiding PMIS trend capture. Original T.MM(F) and Th.MM(F) had near-zero SHAP values (**Figure 3d**), but their combined feature exerted strong positive contributions at high values (SHAP≈10), introducing critical nonlinear information. Similarly, “T4Tcm.MM(F)-T4Tn.MM(F)” had negligible original SHAP values and concentrated distributions around zero (**Figure 3d**), but subtractive interaction yielded large positive/negative SHAP values (≈ ± 10), substantially boosting model contributions. The feature “ln(T.MM(F))-ln(Th.MM(F))” mimicked division via logarithmic subtraction; while its age correlation did not improve, its SHAP value significantly increased, indicating enhanced model impact (**Figure 3e**). In the mitochondrial characteristic ablation experiment, after removing mitochondria-related indicators from 11 key indicators, the remaining 5 indicators (**Table 2**) in the independent HC test set: R^2^ plummeted from 0.809 to 0.437, and MAE increased sharply from 5.687 to 10.274 (**Table 3**). In addition, in our mean-square analysis, the dispersion width of the residuals was basically the same in different prediction age groups, and there was no obvious horn shape, indicating that the prediction stability of the model was equally good in all age groups (**Supplementary Figure 1**; **Supplementary Table 1**). The integrated LightGBM model residual plot shows no systematic bias: the blue LOWESS linefluctuates small and close to zero, proving that the model error is purely random and there is nononlinear systematic bias, that is, it does not suddenly become very inaccurate at a particular age(**Supplementary Figure 2**).

**Table 2:** Ten-fold cross-validation performance of seven regression models on the HC training dataset using the 11 core features.

Model	MAE (mean ± SD) [95%CI]	RMSE (mean ± SD) [95%CI]	R² (mean ± SD) [95%CI]
RandomForest	6.674 ± 0.961 [5.987, 7.361]	9.264 ± 1.319 [8.320, 10.207]	0.768 ± 0.065 [0.722, 0.814]
LightGBM	7.097 ± 0.931 [6.431, 7.763]	9.774 ± 1.248 [8.881, 10.667]	0.742 ± 0.068 [0.693, 0.791]
XGBoost	7.220 ± 0.958 [6.535, 7.905]	10.107 ± 1.474 [9.053, 11.161]	0.723 ± 0.083 [0.663, 0.782]
SVM	15.408 ± 1.231 [14.528, 16.289]	18.609 ± 1.310 [17.672, 19.547]	0.081 ± 0.054 [0.042, 0.120]
KNN	12.817 ± 1.278 [11.903, 13.732]	16.343 ± 1.217 [15.472, 17.213]	0.285 ± 0.110 [0.207, 0.364]
LinearRegression	8.196 ± 1.048 [7.446, 8.946]	10.395 ± 1.138 [9.581, 11.209]	0.709 ± 0.063 [0.665, 0.754]
LASSO	8.352 ± 1.033 [7.613, 9.092]	10.498 ± 1.111 [9.703, 11.293]	0.705 ± 0.056 [0.664, 0.745]
Decision-Tree	8.018 ± 1.002 [7.301, 8.734]	11.596 ± 1.667 [10.403, 12.789]	0.634 ± 0.109 [0.556, 0.712]

**Table 3:** Model performance after ablation of mitochondria-related parameters from the 11 core features.

Model	HC test dataset (n = 87)				TM test dataset (n = 30)			
	MAE	MSE	RMSE	R2	MAE	MSE	RMSE	R2
Random Forest	9.748	149.268	12.218	0.515	13.168	283.017	16.823	-0.889
LightGBM	**10.274**	173.429	13.169	**0.437**	13.671	331.946	18.219	-1.216
XGBoost	10.820	189.154	13.753	0.385	13.318	304.382	17.447	-1.032

It should be added that SHAP analysis quantified feature contributions to the immune aging prediction model, visualizing each parameter’s positive/negative impact on PMIS and its contribution magnitude via color gradients. High values of features with negative SHAP values—such as “ln(T8Tn%)+ln(T8Tcm.MMP%)”, “CD3.MMP%-T8Tn.MMP%”, “T4Tcm.MM(F)-T4Tn.MM(F)”, and “Tc.MMP%+T4Tn.MMP%”—served as core signals delaying immune senescence. Conversely, high values of features with positive SHAP values, including “ln(T4Tem%)+ln(T8Tcm%)”, “MCV+MCHC”, “CD4^+^%+T4Tcm%”, “NK%-NK.MMP%”, and “ln(T.MM(F))-ln(Th.MM(F))”, accelerated immune senescence (**Figures 3d, e**). Each point represents a sample; the vertical axis lists key features, and the horizontal axis denotes SHAP values. Red indicates high feature values, blue low values. Points right of zero reflect positive contributions (higher predicted PMIS), while left points reflect negative contributions (lower predicted PMIS), clarifying feature importance and directional roles in PMIS prediction.

### 3.4 Application of machine learning models

Given the significant age distribution disparities across cohorts, the healthy control (HC) dataset was used as the primary training and test set to enable the model to adequately capture immune signatures in adolescents and young adults and ensure stable generalization within this age range, while the tumor (TM) dataset served as an external validation set to assess predictive robustness in heterogeneous populations. Because of the divergent feature distributions and immune statuses between the TM and HC groups, model accuracy was reduced in the tumor data; however, we hypothesized that the model would yield a higher predicted PMIS than chronological age in cancer patients, indicating accelerated immune aging. Due to the limited training data and lack of subjects over 90 years old, stable trend estimation for the 80-90-year age group was not feasible, so the core validity criterion was defined as the consistent pattern that predicted PMIS exceeds chronological age in cancer patients younger than 80 years. To establish a performance baseline, five regression models (tree-based and linear) were initially trained on raw data with 10-fold cross-validation (10-fold CV) on both HC and TM test sets. When trained solely on the 11 core immune features, all models maintained strong fitting and predictive performance despite the greatly reduced dimensionality (**Table 1**): in the HC test set, mean absolute error (MAE) decreased by approximately 0.99 relative to baselines and R² increased to approximately 0.79, demonstrating efficient and accurate prediction with a compact feature set, and tree-based algorithms (RF, LightGBM, XGBoost) outperformed other methods, supporting further optimization of these three models. To validate the 11 core features, we re-evaluated model performance using the 20 underlying original features; compared with the full set of 72 original features, the 20-feature set reduced redundancy and improved generalization in the HC test set despite a minor drop in cross-validation performance, and the feature interaction and combination strategy further enhanced the predictive capacity of the 11-feature set, which outperformed both the 72-feature and 20-feature sets in cross-validation and independent HC testing. Among the five models, RF performed best on training data while LightGBM performed best on test data: RF reduced cross-validation MAE by approximately 0.63 and LightGBM reduced HC test MAE by approximately 1.30, validating the efficacy of the feature engineering pipeline. We performed hyperparameter optimization via grid search over the parameter space shown in **Table 2**, but in this relatively small cohort, cross-validation–optimized configurations didnot improve out-of-sample performance and even slightly degraded performance in some folds relative to library defaults, while increasing fold-to-fold variance—a sign of overfitting to validation splits—so we retained default hyperparameters for all base learners, with detailed configurations listed in **Supplementary Table 2**.

Finally, the Bagging ensemble strategy was applied to the three top-performing tree-based models for further optimization (**Table 4**); in the HC test set, Bagging improved overall performance and reduced XGBoost MAE by approximately 0.20, and LightGBM exhibited the strongest generalization ability, confirming that ensemble learning effectively enhances the stability and reliability of tree-based models for PMIS prediction.

**Table 4:** Performance of Bagging-ensemble optimized tree-based models on the HC test and TM external test datasets.

Model	HC test dataset (*n* = 87)				TM test dataset (*n* = 30)			
	MAE	MSE	RMSE	R^2^	MAE	MSE	RMSE	R^2^
Random Forest	5.821	62.014	7.875	0.799	12.245	221.619	14.887	-0.479
LightGBM	**5.687**	58.774	7.666	**0.809**	13.075	238.225	15.435	-0.590
XGBoost	5.881	62.435	7.902	0.797	11.320	208.527	14.440	-0.392

### 3.5 Prediction results and group difference analysis

**Figure 4:**
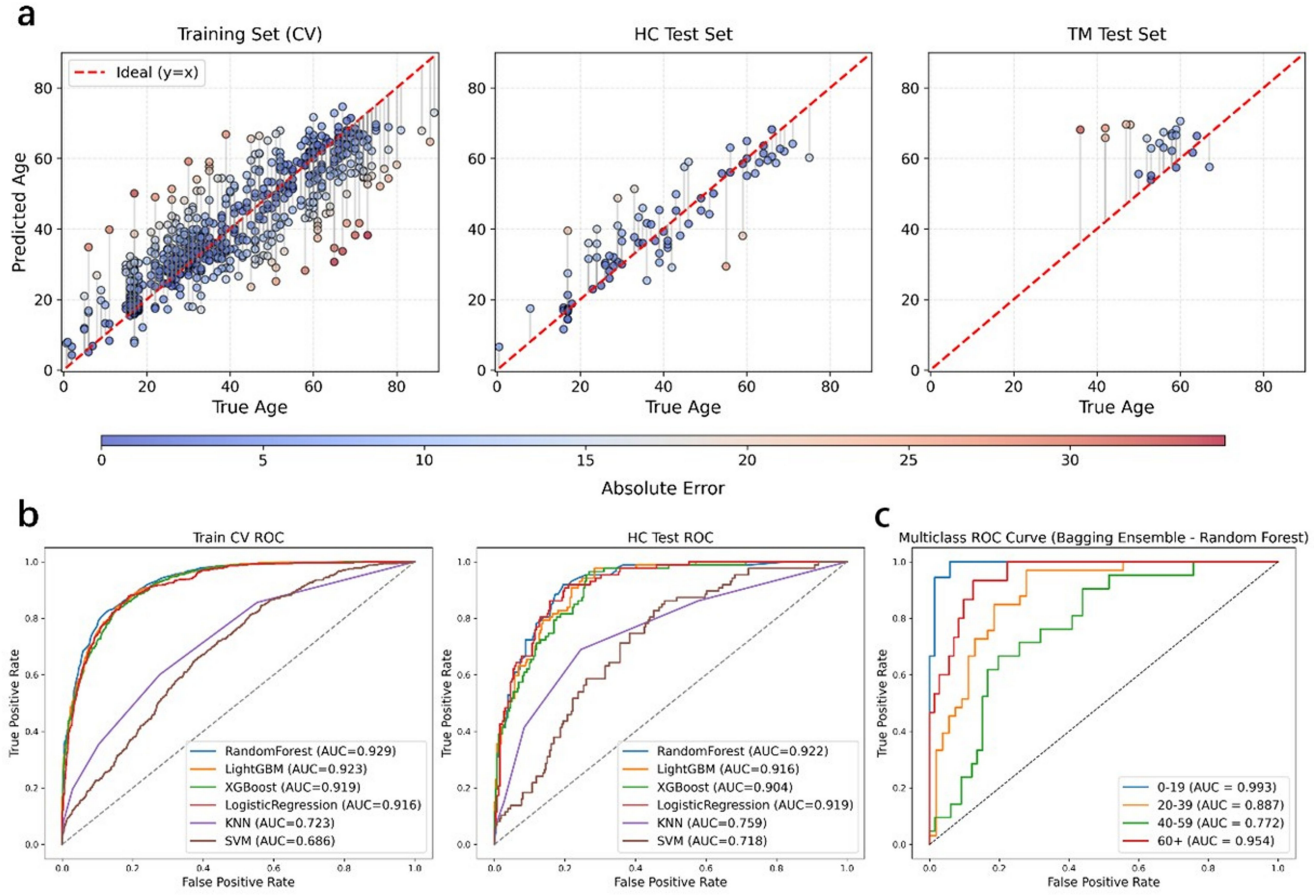
Model performance evaluation and external validation. **(a)** Predicted immune age vs. chronological age in the training set (HC, n=654), HC test set (n=87), and TM external test set (n=30). Red dashed line: y=x (ideal prediction). **(b)** Overall ROC curves and AUC values of six models in the training and HC test sets. **(c)** Multiclass ROC curves and age-stratified AUC of the Bagging-optimized Random Forest model across four age groups (0-19, 20-39, 40-59, ≥60 years). AUC, area under the curve; ROC, receiver operating characteristic; HC, healthy control; TM, tumor patient.

Model performance across populations was assessed using 10-fold cross-validation for PMIS prediction, with results plotted against chronological age (**Figure 4a**; **Table 2**). Models trained on the healthy control (HC) cohort exhibited excellent fitting consistency in both cross-validation and independent testing. Samples from adolescents (~20 years) clustered tightly along the diagonal, reflecting strong predictive performance, whereas the middle-aged group (40–60 years) showed greater dispersion and minor bias likely due to increased physiological heterogeneity. Although predictive accuracy declined slightly in adults over 60 years, predictions remained generally aligned with the diagonal. External validation using the tumor (TM) cohort revealed clear systematic deviation: most TM samples lay above the diagonal, especially among patients under 70 years, confirming that predicted PMIS was significantly higher than chronological age and indicating accelerated immune aging in cancer patients, consistent with biological expectations and supporting model validity. To directly quantify accelerated immune aging, we calculated the immune age gap (Δ =predicted PMIS − chronological age) for each patient. The TM cohort exhibited a strongly positive age gap (median Δ=15.08 years, 95%CI [8.75, 17.41]; one-sided Wilcoxon signed-rank *p* = 3.46e-07; paired *t*-test *p* = 2.94e-08; Cohen’s *d*z = 1.32; rank-biserial *r* = 0.91), with 87% (26/30) of patients classified as immune-older than their chronological age. In contrast, the HC cohort showed a symmetric age gap centered near zero (median Δ=0.92 years, 95%CI [-1.08, 2.54]; *p* = 0.399, n.s.), and the TM gap was significantly larger than the HC gap (Mann-Whitney *U* test, one-sided *p* = 2.40e-8). The negative *R*² value observed in the TM cohort (**Table 1**) reflects this systematic upward offset from the identity line and is therefore biologically interpretable: while *R*² penalizes consistent deviation from *y* = *x*, a directional age gap represents the key signal of accelerated immune aging (**Figure 4a**; **Table 2**). Thus, regression metrics for the TM set are reported for completeness but are not suitable for evaluating the model’s ability to capture immune senescence acceleration.

Samples were stratified into juvenile (0-19), young (20-39), middle-aged (40-59), and elderly (≥60) groups, with ROC curves generated for each. Regression tasks were converted to multi-classification: predictions matching chronological age groups were deemed accurate. Six models (RF, LightGBM, XGBoost, LR, KNN, SVM) were trained on the 11-feature dataset. Cross-validation on HC training/test sets showed RF, LightGBM, XGBoost, and LR achieved AUC >0.9, while KNN and SVM performed poorly (**Figure 4b**). Age-group-specific AUC analysis (**Table 5**) revealed tree models excelled in juveniles (max AUC = 0.93), with LR reaching >0.91, attributed to large sample sizes, concentrated features, and low inter-individual variability. Elderly groups also showed high discrimination (AUC>0.91 for tree/LR models), reflecting consistent age-related immune decline. Young and middle-aged groups performed weaker: young adults-maintained AUC≈0.93 (**Figure 4c**), while middle-aged cross-validation AUC≈0.88 dropped to 0.75–0.79 in independent testing, driven by lifestyle/health/environmental factors increasing immune heterogeneity. Tree and LR models outperformed KNN/SVM; juveniles showed optimal performance, middle-aged adults the weakest. Bagging enhanced the top-performing RF model’s generalization, improving AUC in all age groups except juveniles (already near-perfect), boosting overall stability and discrimination (**Figure 4c**).

**Table 5:** Age-stratified classification performance (AUC) of six models in four age groups.

Model	10-fold CV (training dataset)				HC test dataset			
	0–19	20–39	40-59	≥60	0–19	20–39	40–59	≥60
RF	0.990	0.911	0.812	0.925	0.994	0.893	0.783	0.953
LightGBM	0.989	0.912	0.803	0.910	0.992	0.888	0.799	0.919
XGBoost	0.989	0.906	0.798	0.910	0.992	0.876	0.768	0.907
LR	0.973	0.892	0.817	0.928	0.976	0.881	0.794	0.966
KNN	0.874	0.618	0.597	0.694	0.926	0.737	0.576	0.699
SVM	0.782	0.524	0.606	0.717	0.827	0.476	0.666	0.719

## 4 Discussion

**Figure 5:**
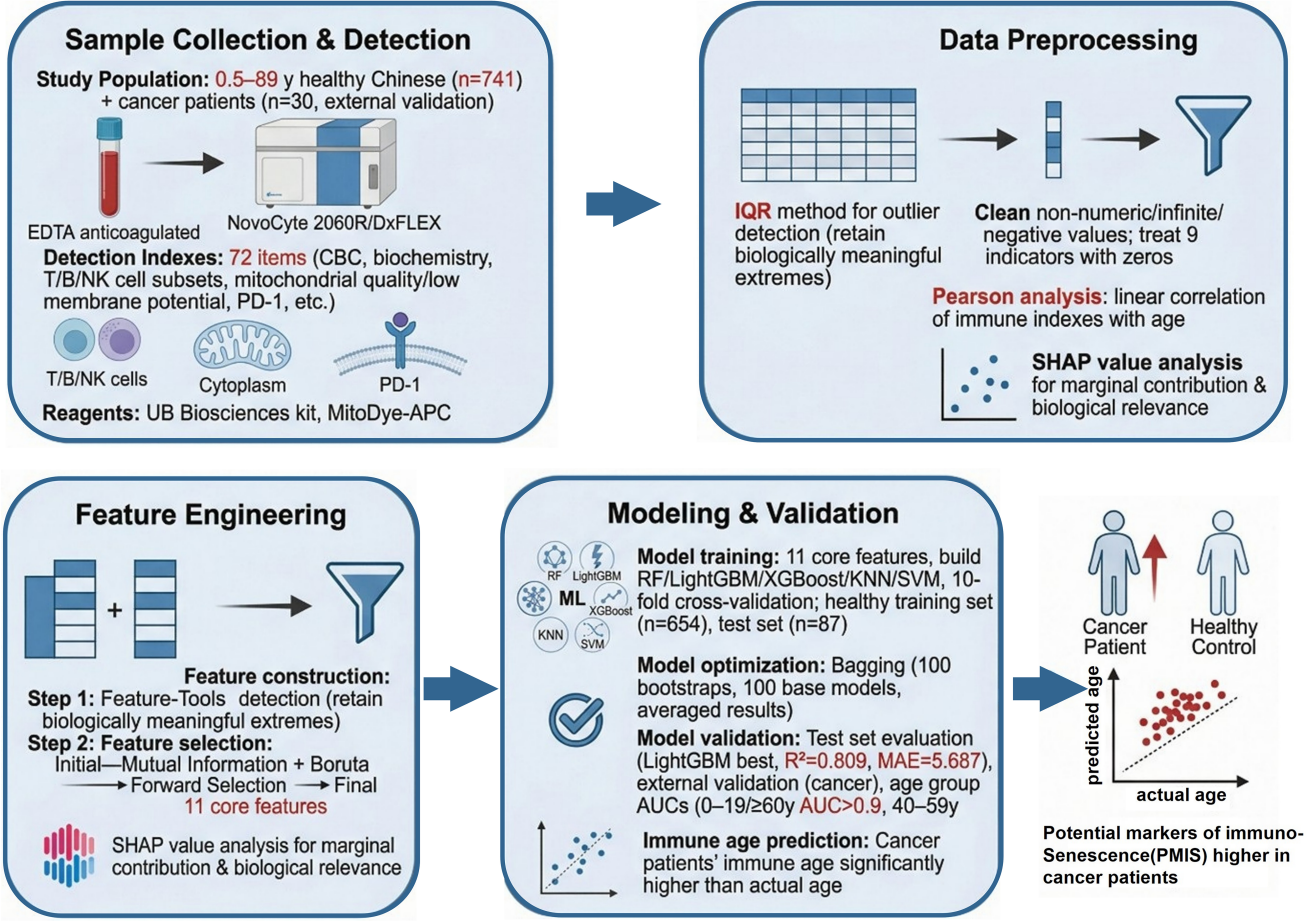
The diagram shows the comprehensive technical route for PMIS calculation and validation. The workflow includes four core stages: sample collection and flow cytometry detection, data preprocessing and quality control, feature engineering and multi-stage screening, machine learning modeling and Bagging optimization, and external validation using tumor samples. The final LightGBM model achieved R²=0.809 and MAE = 5.687 in the HC test set, and successfully identified significantly elevated PMIS in tumor patients. PMIS, potential markers of immunosenescence; LightGBM, light gradient boosting machine; MAE, mean absolute error; R², coefficient of determination.

In this study, comparative analysis of the 13 original important features selected by Boruta and forward selection and the 11 core features including combined features showed that there was a significant overlap in the composition of the two, indicating that the core features mainly came from a few key immune and hematological indicators, such as the cell proportion of T8Tn and the low ratio of mitochondrial membrane potential of T8Tcm. This coincidence suggested that the main explanatory power of the model focused on biologically stable, repeatedly identified features, verifying the robustness of the feature selection process with biological consistency. The PMIS calculation and validation process consists of four core stages: sample collection and flow cytometry detection, data preprocessing and quality control, feature engineering and multi-stage screening, machine learning modeling and Bagging optimization, and external validation using tumor samples. The final LightGBM model successfully identified significantly elevated PMIS in cancer patients with R²=0.809 and MAE = 5.687 in the HC test set (**Figure 5**).

Recent studies have reported that appropriate amount of vitamin K2 can alleviate mitochondrial oxidative stress by activating JNK-1/SIR-2.1/DAF-16 pathway, significantly prolong the lifespan of nematode models, and improve their health status (26). Histone deacetylase 9 (HDAC9) plays an important role in aging-related adipose tissue senescence and mitochondrial dysfunction. In the adipose tissue of mice, HDAC9 expression is positively correlated with age (27). All this suggests that targeting mitochondria is a highly visible marker for aging. In our indexes, MM and MMP^low^% are mitochondria-related indexes. This is supported by a variety of relevant evidence, such as the correlation between these indicators and the therapeutic effect in NSCLC (28), kidney transplant (29), hepatopathy (30), and mental disorders (31) have also been reported. All the above can indicate that such indicators are not empty indicators, but existing clinical detection indicators or the content of relevant reports.

Interestingly, it was worth noting that some variables with low importance and low ranking at the original feature level have significantly improved their prediction contribution after participating in the construction of combined features. For example, the mitochondrial mass of T4Tcm cells and the mitochondrial mass of T4Tn cells were ranked 24th and 25th, respectively in the forward selection, but the two were ranked 5th in the forward selection after subtraction interaction, and the relative importance scores between the features were also improved. The mitochondrial mass of T cells and Th cells ranked 27th and 28th, respectively in the forward selection before the combination, and the relative importance of the combined features rose to sixth place in the forward selection.

From the methodological point of view, this phenomenon suggested that in multi-dimensional immunological data, the marginal effect of a single feature is often limited, and the combination of variables constructed by proper mathematical operation can amplify the potential signal to a certain extent, reflecting the advantage of information fusion across features. This not only demonstrates the rationality of the construction logic of combined features but also reveals the value of feature interactions in modeling complex biological systems. Biologically, the high importance of combinatorial features may reflect processes of coordinated imbalance between different components of the immune system, such as the interaction between T-cell subset proportions and mitochondrial activity or the dynamic coupling of hematologic parameters with immune-metabolic states. These combined signals are closer to the systemic nature of immune senescence and therefore exhibit higher explanatory power in the model. For instance, Tan et al. conducted a study analyzing spatial transcriptomics data of colonic mucosa from healthy controls (HC), patients with Crohn’s disease (CD), and those with ulcerative colitis (UC) (32). Using nonnegative matrix factorization (NMF), they first identified four recurrent cell niches representing functionally distinct tissue microenvironments. Based on these niches, 44 features were systematically constructed to capture three core histopathological dimensions: niche composition, neighborhood enrichment, and niche–gene signaling. A multilayer perceptron (MLP) classifier trained on these features achieved robust performance in both the challenging three-class classification task (HC, UC, CD) and the two-class discrimination of inflammatory bowel disease (IBD) from healthy tissue. Notably, interpretability analyses further revealed that disrupted niche spatial organization served as the most prominent predictor of generalized inflammation, while UC-CD differentiation relied on niche-specific gene expression signatures. This study shares methodological consistency and clinical translational relevance with the present work. Collectively, such research efforts foster mutual validation and cross-application of analytical frameworks, highlighting the potential for synergistic advancement of biomedical representation learning in future investigations. Engaging in this work not only provides a practical solution to the current research challenge but also contributes to integrating the research framework into a broader, generalizable paradigm for principled representation learning in biomedical systems.

The proportion of cell counts in different age groups was relatively stable. There have been reported that T8Tn% can be used for immune monitoring, which is consistent with the conclusion of this study (29). In addition to naive T cell percentage and mitochondrial low membrane potential parameters, the low membrane potential ratio of memory T cells (including Tcm and Tem) contributed more to PMIS. RDW and PCT index gained from blood routine test, which had a greater impact on PMIS in blood routine tests, and the distribution width of red blood cells reflects the uniformity of red blood cell size. These results indicated that the uniformity of red blood cells was greatly affected in the older population or stage, and gradually increased with the increase of age, including anemia, which will affect this parameter and make it difficult to continue to protect or maintain the consistency of cell size. Procalcitonin (PCT) is a marker of bacterial infection. In this study, it was found that the PCT decreased gradually with the increase of age, from 0.25-0.35 in newborns to below 0.23 at 75 years old.

In addition, the change of T cell proportion was little affected by age factors and relatively stable, but the percentage of NK cells gradually increased with age, which was about 20% in newborns and more than 40% in 75-year-old people, and the trend line slope reached about 0.33. This conclusion was consistent with the results of previously published studies (33). Most of the mitochondrial indices showed consistent changes: the mitochondrial mass gradually increased with the increase of age, while the mitochondrial low membrane potential, on the contrary, decreased with the increase of age. In general, the decrease in mitochondrial low membrane potential was slightly higher than the increase in mitochondrial mass. The value of engineered, interaction-aware representations is increasingly recognized across biomedical machine learning, and our findings align with this broader direction. In a conceptually related study, Tan et al. analyzed spatial transcriptomics of colonic mucosa from healthy controls and patients with Crohn’s disease or ulcerative colitis (32). Rather than modeling individual genes, they used non-negative matrix factorization to define four recurrent cellular niches and then engineered 44 higher-order features describing niche composition, neighborhood enrichment, and niche–gene signaling; a multilayer-perceptron classifier used these to separate inflammatory bowel disease from healthy tissue and to distinguish the two disease subtypes, and their interpretability analysis showed that disruption of niche spatial organization, rather than any single molecular feature, was the strongest predictor of inflammation. The parallel to the present work is methodological: in both cases the discriminative signal resides not in raw measurements but in engineered features that encode interactions between components—spatial niche relationships in their setting, and arithmetic combinations of T-cell subset proportions with mitochondrial readouts in ours. This mirrors our central observation that variables with negligible individual SHAP importance (e.g. T4Tcm.MM(F) and T4Tn.MM(F)) become among the most influential predictors once combined into interaction terms (**Figures 3d, e**), and that both pipelines depend on *post-hoc* interpretability—their feature-attribution analysis and our SHAP decomposition—to recover biological meaning from the engineered feature space. Viewed together, these studies position heuristic, domain-guided feature construction as a step toward more principled, interaction-aware representation learning for clinical prediction. A natural extension of our framework is therefore to move beyond peripheral-blood, cell-intrinsic measurements toward spatially resolved data: applying the same combination-and-selection logic to spatial transcriptomic or imaging-based immune profiling would allow PMIS to capture not only the composition and metabolic state of immune cells but also their spatial organization and cell–cell communication, yielding a richer, context-aware characterization of immune aging.

This study revealed age-related predictive biomarkers for immune parameters, including mitochondrial low membrane potential and mitochondrial mass. These blood-derived immune indexes related to age have the basis and feasibility for the calculation of PMIS. This study was a retrospective study. The samples were taken at baseline and were all healthy. We found potential predictive markers, the complex AutoGluon modeling approach avoids overfitting. However, without external datasets, our study was not yet able to assess whether these models can be extended to screen out better represented coves in the physical examination population, which remained a major limitation of the study. Another major limitation is the issue of age distribution, such as the under-distribution of the elderly population. Moreover, there is bias in the cancer cohort. The larger, age-matched validation cohorts are required for robust disease-related conclusions, which is planned as future work. As for the analysis and consistency evaluation of various ML algorithms, we believe that the results of different ML models of PMIS are consistent, which is objective and advanced to a certain extent. It was expected to expand the data and upgrade the learning algorithm for this kind of data, and it would be widely used in physical examination and medical direction.

In this study, peripheral blood was used to explore quantitative indicators related to immunosenescence. The age-related aging characteristics were numerically reflected by immune cell indexes and mitochondrial indexes. Through external verification of tumor samples, we verified the high PMIS caused by accelerated aging. In fact, we did get such results, which provide a new idea for the judgment of aging-related diseases in the future. In the future, the model is trained on chronological age and requires prospective clinical validation (frailty, infection risk, mortality) as a major limitation and future work. In the future, hippocampal metabolic analysis, confocal microscopy and other methods will be used to verify the mitochondrial results to compensate for the possible problems of single flow cytometry methodology. And we will expand immune indicators and conduct studies with larger samples, obtain more effective calculation methods through systematic and rigorous analysis, and gradually apply them to the diagnosis and treatment of clinical diseases.

## Data Availability

The raw data supporting the conclusions of this article will be made available by the authors, without undue reservation.

## Supplementary material

Supplementary Figure 1 Calibration curve of ensembled LightGBM model. The fitted regression line in orange, with a 95% confidence band, is close to the black y=x ideal diagonal. This indicates that the immune age predicted by the model is highly consistent with the true physiological age in the overall distribution, and no serious systematic overestimation or underestimation occurs. In the younger age group (<30 years), the orange line was slightly higher than the black line. At higher ages (>65 years), the orange line is slightly lower than the black line. In the world of aging clocks, this phenomenon, known as “Regression to the mean,” is a classic and normal feature of epigenetic and immune clocks. This proves that the model captures true biological regularities and is not forced to remember the data due to overfitting.

Supplementary Figure 2 residual plot of ensembled LightGBM model. In the analysis of the residual distribution plot, the green residual was roughly evenly and randomly scattered on both sides of the zero-error line (y=0, red line). The blue LOWESS smooth trend line almost coincides with the red line.

Supplementary Table 1 Robust ranking of predictors of immune senescence. To assess feature stability, 100 bootstrap iterations using 90% random sampling were executed on the training set. Within each iteration, the top 11 features were strictly filtered by combining the Boruta algorithm with importance score rankings. The consistently high selection frequency of these 11 core features under data perturbation indicates their reliability as predictors of immune aging.

Supplementary Table 2 Hyperparameters of machine learning models (default settings).
