# Supplementary figures and images for: A novel study to calculate immune-aging from peripheral blood T lymphocyte subsets and their mitochondrial parameters in healthy Chinese subjects
Source: Front Immunol. 2026 Jul 7;17:1857636. doi: 10.3389/fimmu.2026.1857636 (PMC13386123; doi:10.3389/fimmu.2026.1857636)

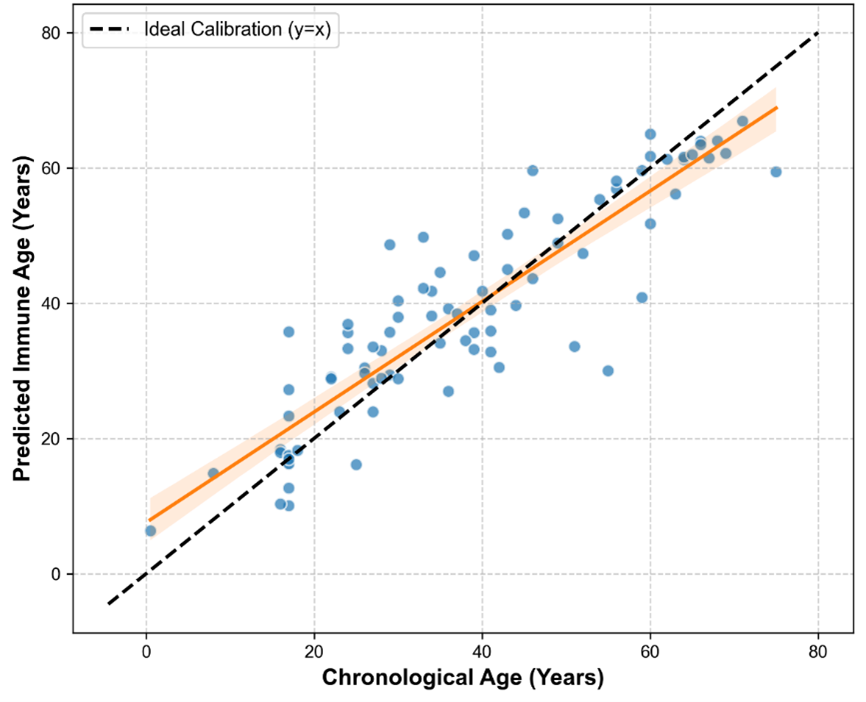

Supplement: Supplementary Figure 1 — Calibration curve of ensembled LightGBM model. The fitted regression line in orange, with a 95% confidence band, is close to the black y=x ideal diagonal. This indicates that the immune age predicted by the model is highly consistent with the true physiological age in the overall distribution, and no serious systematic overestimation or underestimation occurs. In the younger age group (<30 years), the orange line was slightly higher than the black line. At higher ages (>65 years), the orange line is slightly lower than the black line. In the world of aging clocks, this phenomenon, known as “Regression to the mean,” is a classic and normal feature of epigenetic and immune clocks. This proves that the model captures true biological regularities and is not forced to remember the data due to overfitting. [file Image1.tif]

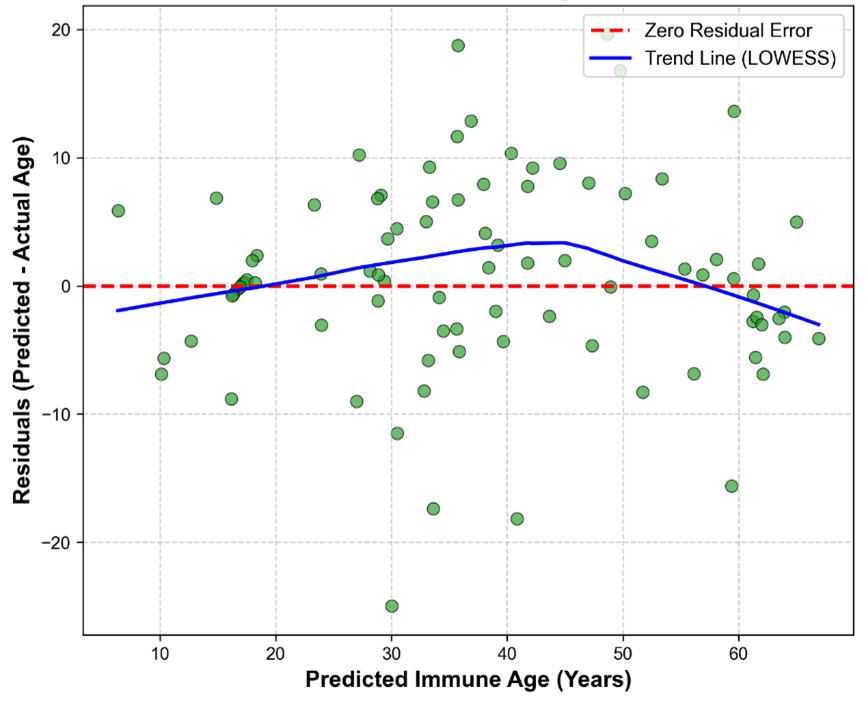

Supplement: Supplementary Figure 2 — residual plot of ensembled LightGBM model. In the analysis of the residual distribution plot, the green residual was roughly evenly and randomly scattered on both sides of the zero-error line (y=0, red line). The blue LOWESS smooth trend line almost coincides with the red line. [file Image2.tif]
