# Supplementary material for: A novel study to calculate immune-aging from peripheral blood T lymphocyte subsets and their mitochondrial parameters in healthy Chinese subjects
Source: Front Immunol. 2026 Jul 7;17:1857636. doi: 10.3389/fimmu.2026.1857636 (PMC13386123; doi:10.3389/fimmu.2026.1857636)
Supplement: Supplementary Table 1 — Robust ranking of predictors of immune senescence. To assess feature stability, 100 bootstrap iterations using 90% random sampling were executed on the training set. Within each iteration, the top 11 features were strictly filtered by combining the Boruta algorithm with importance score rankings. The consistently high selection frequency of these 11 core features under data perturbation indicates their reliability as predictors of immune aging. [file DataSheet1.pdf]

Supplementary Table 1 Robust ranking of predictors of immune senescence.

To assess feature stability, 100 bootstrap iterations using 90% random sampling were executed on the training set. Within each iteration, the top 11 features were strictly filtered by combining the Boruta algorithm with importance score rankings. The consistently high selection frequency of these 11 core features under data perturbation indicates their reliability as predictors of immune aging.

Feature	Stability (%)
ln(T8Tn%)+ln(T8Tcm.MMP%)	100.0
ln(T4Tem%)+ln(T8Tcm%)	100.0
CD3.MMP%-T8Tn.MMP%	100.0
MCV+MCHC	96.0
$CD4^{+}$%+T4Tcm%	94.0
T4TN%	93.0
ln(T.MM(F))-ln(Th.MM(F))	93.0
T4Tcm.MM(F)-T4Tn.MM(F)	90.0
NK%-NK.MMP%	87.0
Ts.MMP%+T4Tn.MMP%	87.0
PLT	81.0
