# Supplementary material for: A novel study to calculate immune-aging from peripheral blood T lymphocyte subsets and their mitochondrial parameters in healthy Chinese subjects
Source: Front Immunol. 2026 Jul 7;17:1857636. doi: 10.3389/fimmu.2026.1857636 (PMC13386123; doi:10.3389/fimmu.2026.1857636)
Supplement: Supplementary Table 2 — Hyperparameters of machine learning models (default settings). [file DataSheet2.pdf]

Supplementary Table 2 Hyperparameters of machine learning models (default settings).

Model	Final hyperparameters used (library defaults; random_state=42)
Random Forest (sklearn RandomForestRegressor)	n_estimators=100, criterion='squared_error', max_depth=None, min_samples_split=2, min_samples_leaf=1, max_features=1.0, bootstrap=True
LightGBM (LGBMRegressor)	boosting_type='gbdt', n_estimators=100, learning_rate=0.1, num_leaves=31, max_depth=-1, min_child_samples=20, subsample=1.0, colsample_bytree=1.0, reg_alpha=0.0, reg_lambda=0.0
XGBoost (XGBRegressor)	n_estimators=100, learning_rate=0.3, max_depth=6, subsample=1.0, colsample_bytree=1.0, gamma=0, min_child_weight=1, reg_alpha=0, reg_lambda=1
Ensemble wrapper (Bagging)	100 base estimators, sample_frac=0.9, replace=True (bootstrap), predictions averaged
